# The role of SIRT1-FXR signaling pathway in valproic acid induced liver injury: a quantitative targeted metabolomic evaluation in epileptic children

**DOI:** 10.3389/fphar.2024.1477619

**Published:** 2024-11-07

**Authors:** Mingming Zhao, Guofei Li, Limei Zhao

**Affiliations:** Department of pharmacy, Shengjing Hospital of China Medical University, Shenyang, Liaoning, China

**Keywords:** valproic acid, hepatotoxicity, metabolomics, mechanism, SIRT1, FXR

## Abstract

**Aim:**

This study aimed to gain deeper insights into the hepatotoxicity mechanisms of valproic acid (VPA), as well as to identify potential risk markers for VPA-induced hepatotoxicity.

**Methods:**

Twenty-two children with epilepsy treated with VPA monotherapy were divided into a normal liver function (NLF) group, a mild abnormal liver function (ANLF1) group, and a serious abnormal liver function (ANLF2) group based on their liver function indicator levels. The full quantitative targeted metabolomics technique was used to systematically investigate how the differential endogenous metabolic components change with the development of liver injury.

**Results:**

A total of 195 metabolic components were quantitatively analyzed. Nineteen identified metabolites, including five organic acids, four short-chain fatty acids, four amino acids, three fatty acids, and three benzenoids, differed significantly among the three groups, showing a strong association with VPA-induced hepatotoxicity. Only three bile acid metabolites, taurodeoxycholic acid, taurochenodeoxycholic acid, and deoxycholic acid, were significantly different between the ANLF1 and ANLF2 groups, increasing at first and then decreasing with the aggravation of liver injury. The mechanistic evaluation showed that SRT1720 activation could alleviate the severity of liver function abnormalities induced by VPA. Immunocoprecipitation indicated that VPA significantly increased the acetylation level of FXR, and the application of agonist SRT1720 can antagonize the acetylation of FXR by VPA.

**Conclusion:**

Nineteen identified metabolites showed a strong association with hepatotoxicity and three bile acid metabolites changed with the development of liver injury. The SIRT1–FXR pathway was firstly proposed to participate in VPA-induced hepatotoxicity.

## 1 Introduction

Valproic acid (VPA) is an anticonvulsant drug widely used to treat various types of epilepsy as monotherapy or adjunctive therapy (Perucca, 2002; Ghodke-Puranik et al., 2013). VPA is well tolerated but may lead to fatal hepatotoxicity, potentially limits its clinical application (Eadie et al., 1988; Powell-Jackson et al., 1984; Schmid et al., 2013). Since its introduction, the mechanism of hepatotoxicity induced by VPA has been widely studied, but its exact molecular mechanism has not yet been identified.

It has been reported that the formation of reactive unsaturated metabolites that accumulate in hepatocytes, playing a critical role in the pathogenesis of hepatotoxicity (Rettenmeier et al., 1986; Tang and Abbott, 1996). VPA and its metabolites can lead to liver toxicity by inhibiting mitochondrial β-oxidation, reducing antioxidant capacity, promoting hepatic steatosis, and so on (Chang and Abbott, 2006; Guo et al., 2019; Silva et al., 2008). VPA is a simple branched-chain fatty acid, but its metabolism is a complicated process involving multiple pathways (Ghodke-Puranik et al., 2013). Thus, the mechanisms of hepatotoxicity induced by VPA are extremely complex and sophisticated, and some of them are still poorly understood. Considering that VPA-induced hepatotoxicity involves multiple pathways and mechanisms, it may be not related to a single factor but a variety of factors such as environment, genome, age, and disease state. Nevertheless, its final effect will be reflected in its metabolic profile, so it is of great significance to investigate this further.

At present, metabolomics as an emerging “omics” science has become a vital tool for drug discovery and therapeutics. Unlike genomics, transcriptomics or proteomics, which indicate what might happen, metabolomics indicates what is currently taking place (Wishart, 2016). In particular, metabolomics represents not only the endogenous factors but also the environmental factors (Houten, 2009)^.^ In this regard, application of metabolomics towards the complex hepatotoxicity induced by VPA may provide unique insights into the mechanism and strategies for its safe use in clinical settings. However, the literature on this research topic is limited, and detailed characteristics of VPA‐treated epileptic children, who are at high risk for developing hepatotoxicity, remain unknown. Moreover, no research has focused yet on the dynamic change of VPA-induced liver injury. Therefore, it is of great importance to investigate whether there are related endogenous metabolites changing with the degree of liver function in different periods of the occurrence and development of liver injury.

As important signaling molecules, bile acids play an important role in glucose metabolism, lipid metabolism and energy metabolism by activating different bile acid receptors, such as farnesoid X receptor (FXR), vitamin D receptor, pregnane X receptor and G protein-coupled bile acid receptor (Cai et al., 2022; Qi et al., 2015). Therefore, it is very important to maintain the metabolic balance of bile acids. Multiple studies have shown that abnormal bile acid metabolism may be one of the early events of liver damage (Yamazaki et al., 2013).

FXR has been recognized as a major bile acid sensor and an important regulator of bile acid synthesis, metabolism and transport. It was reported that OA inhibited FXR expression and activity by downregulating SIRT1, leading to disturbances in bile acid metabolism as well as liver injury (Liao et al., 2023). However, the relationship between bile acid profiles and VPA hepatotoxicity has not been explored.

To obtain more insights into the hepatotoxicity mechanisms of VPA as well as to identify potential risk and markers, we performed the full quantitative targeted metabolomics study based on Q300™ to systematically investigate how the differential endogenous metabolic components change with the development of liver injury in children exhibiting VPA-induced hepatotoxicity. This metabolomic research may shed light on the mechanism of VPA-induced hepatotoxicity and provide new strategies for a clinically safe application of VPA.

## 2 Materials and methods

### 2.1 Metabolomic profiling in epileptic children

#### 2.1.1 Study population and serum sample collection

This study was approved by the Ethics Committee of Sheng Jing Hospital, China Medical University (Shenyang, China), and written informed consent was provided by the parents of all the participating children. Twenty-two children with epilepsy treated with VPA monotherapy at the Department of Neurology participated in this study. All the subjects were diagnosed with symptomatic epilepsy according to the etiologic classification of epilepsy and took VPA as a monotherapy for 2 months. The age, body weight, and liver function indicators [aspartate transaminase (AST) and alanine transaminase (ALT)] of each patient were recorded. Based on their liver function test results, the subjects were divided into three groups: (1) normal liver function (NLF) group, in which all indicator levels were lower than the upper reference limits, (2) serious abnormal liver function (ANLF2) group, in which ALT ≥3 × upper limit of normal (ULN), and/or AST ≥3 × ULN, and (3) mild abnormal liver function (ANLF1) group, in which 3 × ULN ≥ ALT ≥2 × ULN, and/or 3 × ULN ≥ AST ≥2 × ULN.

Peripheral blood samples were collected from all the subjects just before the last maintenance dose was administered. The serum samples were centrifuged at 4000 rpm for 10 min, and immediately stored at – 80 °C for determination.

#### 2.1.2 Sample preparation

The sample preparation was performed by Metabo-Profile (Shanghai, China). Briefly, 25 μL of serum was added to a 96-well plate, which was transferred to the Biomek 4000 workstation (Biomek 4000, Beckman Coulter, Inc., Brea, California, United States). Then, 100 μL ice cold methanol with partial internal standards was automatically added to each sample and vortexed vigorously for 5 min. The plate was centrifuged at 4000 *g* for 30 min (Allegra X-15R, Beckman Coulter, Inc., Indianapolis, IN, United States). The plate was subsequently returned to the workstation. Afterwards, 30 μL of the supernatant was transferred to a clean 96-well plate, after which 20 μL of a freshly prepared derivative reagent was added to each well. The plate was sealed, and the derivatization was carried out at 30°C for 60 min. After derivatization, 350 μL of ice cold 50% methanol solution was added to dilute the sample. The plate was stored at −20°C for 20 min, followed by 4000 g centrifugation at 4°C for 30 min. After this, 135 μL of the supernatant was transferred to a new 96-well plate with 15 μL internal standards in each well. Serial dilutions of derivatized stock standards were added to the left wells. Finally, the plate was sealed for ultra-performance liquid chromatography coupled to a tandem mass spectrometry (UPLC-MS/MS) analysis.

The pooled quality control (QC) samples were prepared by mixing aliquots of the study samples so that the samples could broadly represent the biological average of the whole sample set. The QC samples for this project were prepared with the test samples and injected at regular intervals (after every 10 test samples for UPLC-MS/MS) throughout the analytical run.

#### 2.1.3 Targeted metabolomic profiling and data processing

Ultra-performance liquid chromatography coupled to a tandem mass spectrometry (UPLC-MS/MS) system (ACQUITY UPLC-Xevo TQ-S, Waters Corp., Milford, MA, United States) was used to quantitate the metabolites in this study. The endogenous metabolites were separated on an ACQUITY UPLC BEH C_18_ column (1.7 μm, 2.1 × 100 mm) with gradient elution using a mobile phase consisting of 0.1% formic acid (A) and acetonitrile(B) at a flow rate of 0.4 mL min^-1^. The gradient conditions were as follows: 0–1 min (5% B), 1–12 min (5%–80% B), 12–15 min (80%–95% B), 15–16 min (95–100%B), 16–18 min (100%B), 18–18.1 min (100%–5% B), 18.1–20 min (5% B). The samples were analyzed using an electrospray probe in the negative ionization mode with the voltage set at −2.0 kV and in the positive ionization mode with the voltage set at 1.5 kV. The source temperature was set at 150 °C and the desolvation temperature was 550 °C. The raw data files generated by UPLC-MS/MS were processed using MassLynx software (V4.1, Waters, Milford, MA, United States) to perform peak integration, calibration, and quantitation for each metabolite.

#### 2.1.4 Statistical analysis

The multivariate and univariate statistical analyses of the obtained data were carried out using the R software package (http://cran.r-project.org/). The multivariate statistical analysis was performed by principal component analysis (PCA), partial least squares discriminant analysis (PLS-DA) and orthogonal partial least-squares discriminant analysis (OPLS-DA). Student’s *t*-test and Mann-Whitney U test were used to compare the differences between two groups. One-way univariate analyses of variance (ANOVA) and Kruskal–Wallis H test were used among multiple groups. In all the analyses, the results were considered statistically significant at a *p*-value <0.05. Moreover, metabolites with variable importance on projection (VIP) > 1, Fold change (FC, |log2FC|)> 0, and *p* < 0.05 were counted as critical.

### 2.2 Mechanistic evaluation of SIRT1/FXR signaling pathway

#### 2.2.1 Materials

Valproate Sodium (≥98%, HPLC) was purchased from Sigma-Aldrich Chemical Co. (St. Louis, MO, United States) and SRT1720 was from Toronto Research Chemicals Inc. (Toronto, ON, Canada). Antibodies against CYP7A1, CYP8B1, FXR, SHP, β-actin, SIRT1 and anti-acetylated-lysine antibodies were purchased from Cell Signaling Technology Inc. (Danvers, MA), Proteintech Group (Chicago, IL) and Abcam Biotechnology (Massachusetts, United States). Horseradish peroxidase-labeled goat anti-rabbit IgG and rabbit anti mouse IgG were from Abbkine Scientific Co. Ltd. (Wuhan, China).

#### 2.2.2 Cell culture and treatment

The HepG2 cell line (Shanghai Institute of Biological Sciences, Chinese Academy of Science, Shanghai, China) was maintained in RPMI-1640 containing 10% (v/v) fetal bovine serum (FBS) and 1% (v/v) penicillin/streptomycin in a 5% CO_2_ humidified 37°C incubator. Cells were then exposed to the solution (group A), 2 mM VPA (group B), 5 mM SIRT1720 (group C), 5 mM SIRT1720 and 2 mM VPA (group D).

#### 2.2.3 Cytotoxicity assay and biochemical analysis

When the cell density was about 90%, the cells were first counted, and then the cell suspension were seeded in a 96-well cell culture plate. Then, the cells were cultured overnight and treated with different drugs according to the experimental group. Five replicates were made for each measurement. After the treatment, the culture plates were incubated in 37°C and 5% CO_2_ incubator for 24 h, 48h and 72 h respectively for CCK-8 detection. ALT and AST activities, and the concentrations of total cholesterol and triglycerides were measured by the commercial kits according to the kit instructions (Nanjing Jiancheng Biology Engineering, Nanjing, China). The oil red O staining was carried out for histopathology.

#### 2.2.4 RNA extraction and real-time PCR

Total RNA was extracted using the simple RNA Extract kit (Tiangen Biotech, Beijing, China) according to the manufacturer’s protocol and then transcribed into complementary DNA (cDNA) by reverse transcription using the PrimeScript RT Reagent Kit (TaKaRa, Osaka, Japan). Quantitative PCR (qPCR) was carried out by an ABI Prism 7500 system (Applied Biosystems, Foster City, CA, United States). The expression of the genes was determined relative to β-actin as an internal control, and the relative abundance was calculated using the 2^−ΔΔCT^ method. The primer sequences are listed in Supplementary Table S1.

#### 2.2.5 Western blot analysis

Western blot analysis was performed using standard procedures. Total proteins were extracted from the cells using RIPA buffer with protease inhibitors and the protein concentration was measured using the BCA protein assay kit. Proteins were separated on the SDS-PAGE with 10% (w/v) polyacrylamide gels and transferred to the polyvinylidene fluoride (PVDF) membranes. Then the membranes were incubated with the following antibodies (CYP7A1 CYP8B1 FXR SHP SIRT1 and β-actin) overnight at 4°C. Horseradish peroxidase-conjugated secondary antibodies were used to stain the membranes and enhanced chemiluminescence using the Amersham Imager 600.

#### 2.2.6 Coimmunoprecipitation analysis

Coimmunoprecipitation was carried out using an immunoprecipitation/Coimmunoprecipitation kit (#A10022, Abmart.) following the manufacturer’s protocol. Briefly, 200 μL of total protein lysates (the concentration was adjusted to 1 μg/μL) was first cleared with 60 μL of 50% protein A agarose beads by centrifugation. And then the extract was incubated with 2 μL specific antibodies as indicated at 4°C overnight. The antigen-antibody complexes were captured using protein A agarose beads and then washed, boiled and subjected to SDS-PAGE and Western blotting.

#### 2.2.7 Data analysis

The data were presented as the means ± SD and statistical analyses were performed using the statistical package for social science (SPSS) software (version 24.0; SPSS Inc., IL, United States). Normal distribution and homogeneity of the variances were tested firstly. And then the parameters were compared by Students’ independent sample *t*-test or 1-way analysis of variance. Statistical significance was defined by a two-tailed *p*-value <0.05.

## 3 Results

### 3.1 Demographics of the study population

The demographic data and clinical characteristics of the 22 epileptic children enrolled in this study are summarized in Table 1, with seven cases in the ANLF1 group, seven cases in the ANLF2 group, and eight cases in the NLF group. There was no significant difference in age, sex, height, weight, and daily dose of VPA among the three groups. The concentrations of serum ALT and AST in the ANLF2 group were significantly higher than those in the ANLF1 group and NLF groups (*p* < 0.05).

**TABLE 1:** Characteristics of studied subjects in serum targeted metabolomic analysis.

Characteristics	ANLF2 group (n = 7)	ANLF1 group (n = 7)	NLF group (n = 8)	*p*-Value
Age	3.00 (1, 11)	7.00 (6, 10)	5.50 (3.25,9.50)	0.336
Sex (M/F)	5/2	5/2	4/4	—
Height (cm)	112.14 ± 39.19	129.14 ± 23.67	125.13 ± 26.58	0.397
Weight (kg)	24.57 ± 19.63	34.14 ± 12.60	28.13 ± 11.47	0.152
Daily VPA Dose (mg/kg)	17.25 ± 8.45	15.57 ± 3.63	16.79 ± 5.90	0.955
VPA concentration(μg/mL)	60.74 ± 23.65	62.04 ± 18.66	60.09 ± 11.23	0.867
CDR_VPA_(μg/mL per mg/kg)	4.23 ± 2.50	4.20 ± 1.84	3.84 ± 1.06	0.900
ALT (U/L)	136.71 ± 19.80	75.14 ± 17.78	23.25 ± 5.85	0.000*
AST (U/L)	125.00 ± 51.05	63.43 ± 27.73	22.50 ± 6.19	0.000*

### 3.2 Serum metabolomic profiling

#### 3.2.1 Profile of metabolic components

A total of 195 metabolites in serum samples of 22 children with epilepsy were quantitatively analyzed in this study based on Q300™ technology. As shown in Supplementary Figure S1, the 195 endogenous metabolites comprised 41 amino acids, 40 fatty acids, 35 organic acids, 21 bile acids, 14 phenyl derivatives, 14 sugars, 12 carnitines, eight short-chain fatty acids, five phenylpropionic acids, and five other categories. To ensure accuracy, reliability, and stability of the metabolic analysis, QC samples were interspersed throughout the analytical process. The heat map analysis and PCA plot of the QC samples are shown in Supplementary Figure S2. The QC samples were close to each other and aggregated well, indicating that the stability of the instrument was good and the determination of the metabolites was accurate.

#### 3.2.2 The multivariate statistical analyses

**FIGURE 1:**
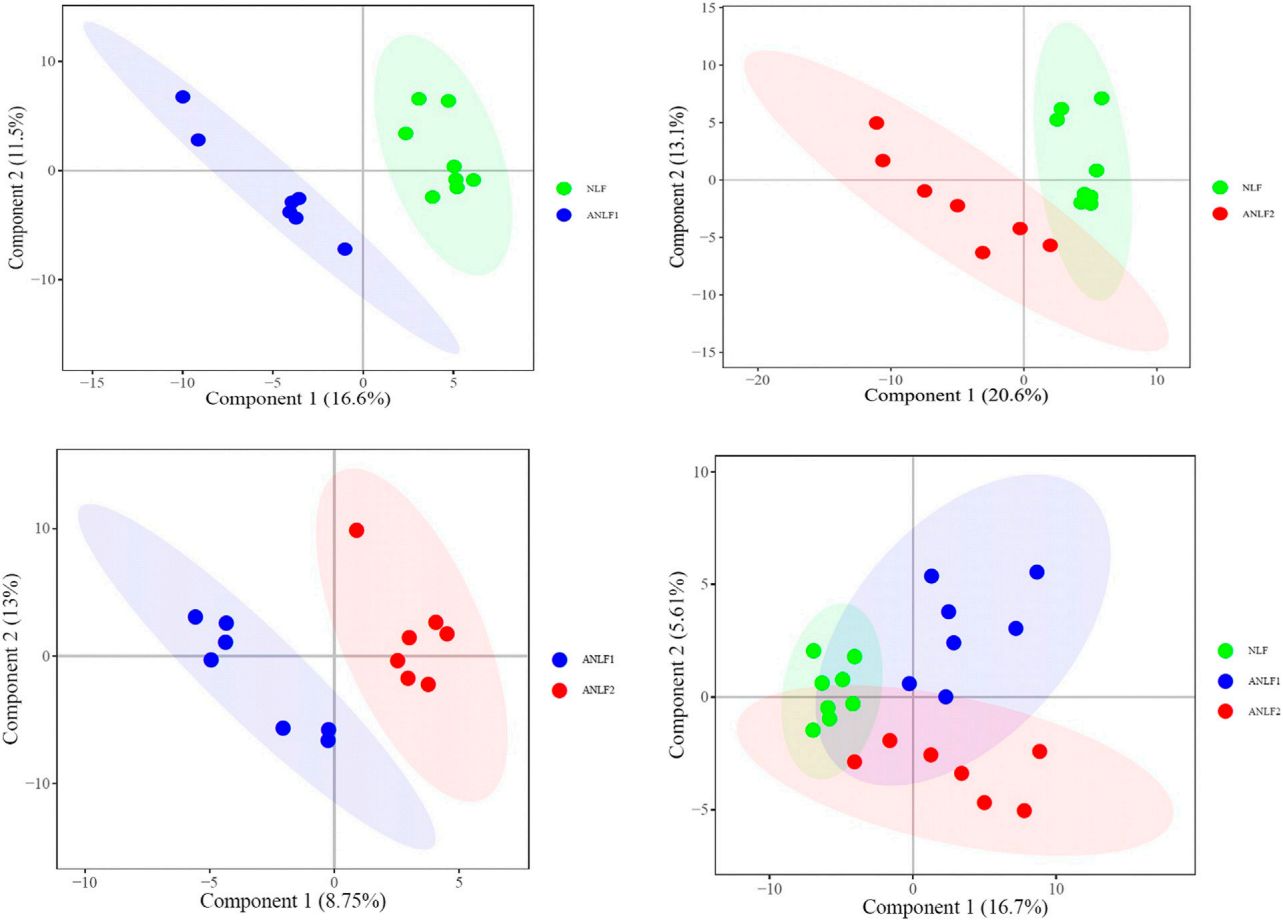
Score plots of the PLS-DA model of the serum of children with epilepsy in three groups of ANLF1, ANLF2 and NLF.

**FIGURE 2:**
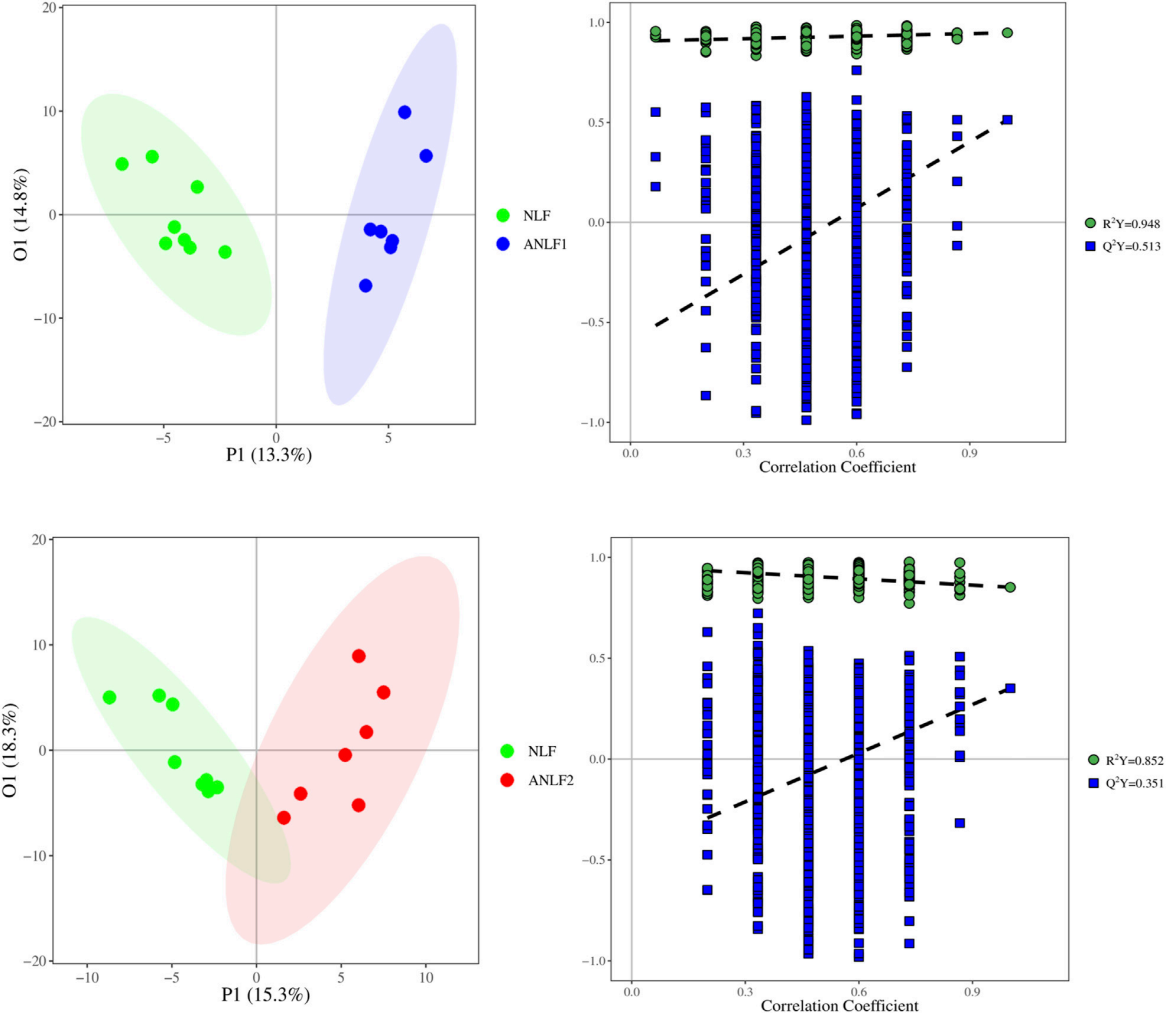
Score plots of the OPLS-DA model of the serum of children with epilepsy between the ANLF1 or ANLF2 group and NLF group.

First, a PLS-DA model was constructed to analyze the systemic differences in the serum of children with epilepsy in the ANLF1, ANLF2 and NLF groups. As shown in Figure 1, there was an apparent separation between each two groups and among all three groups. Then, the metabolic patterns were further analyzed by an OPLS-DA model to characterize the changes between the ANLF1 or ANLF2 group and the NLF group, which are displayed in Figure 2. The OPLS-DA score plots revealed a visible separation of the ANLF1 and ANLF2 groups from the NLF group. Based on the results of OPLS-DA model, the VIP value was used to characterize the metabolites associated with VPA-induced hepatotoxicity.

#### 3.2.3 The univariate statistical analyses

Subsequently, the univariate analysis was carried out through independent sample *t*-test and ANOVA with |log2FC|> 0 and *p* < 0.05. As Supplementary Figure S3 shows, 35 changeable metabolites between the NLF and ANLF1 group, 32 metabolites between the NLF and ANLF2 group, and three metabolites between the ANLF1 and ANLF2 group were identified.

#### 3.2.4 Differential metabolic component screening

**FIGURE 3:**
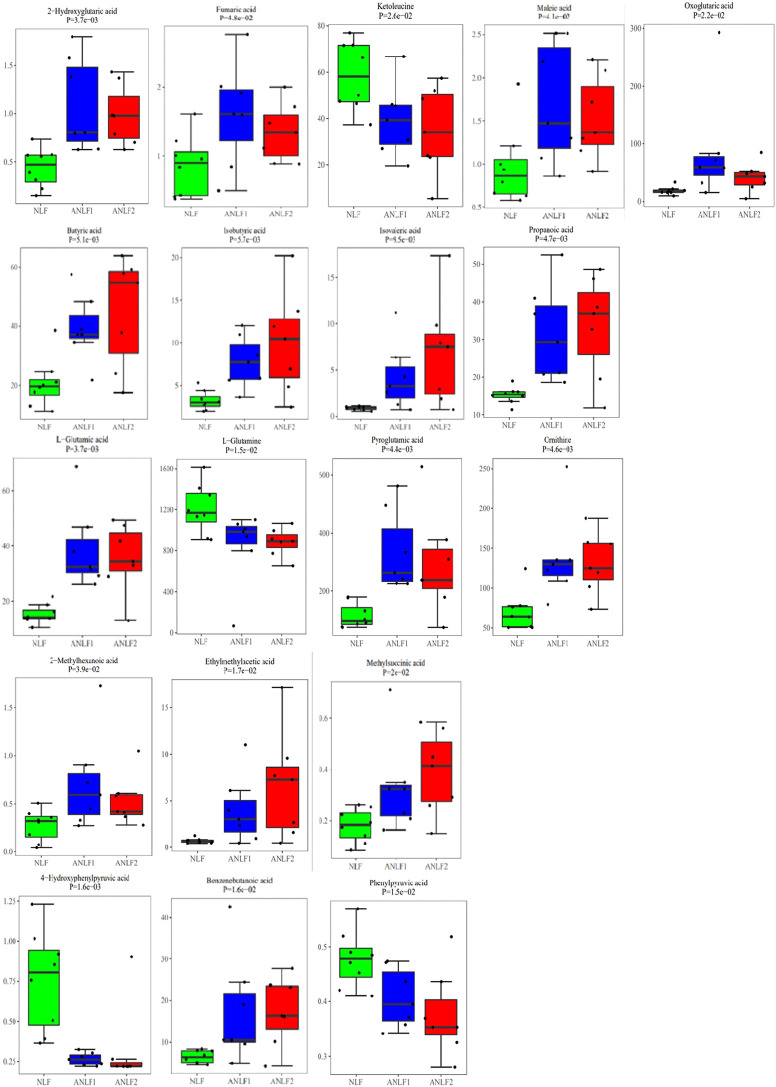
Box plots of the 19 metabolites among the three groups.

Combining with the analysis by using multivariate and univariate statistical methods, nineteen metabolites were shown to differ significantly among the groups and had a strong association with VPA-induced hepatotoxicity, including five organic acids, four short-chain fatty acids, four amino acids, three fatty acids, and three benzenoids. Table 2 provides the corresponding *p* values, fold changes, and VIP scores of these 19 metabolites, while Figure 3 shows the concentrations of each metabolite among the three groups.

**TABLE 2:** Identification of the most significantly changed metabolites among the three groups of ANLF1, ANLF2 and NLF.

Metabolites	Class	*p*	|log2FC|	VIP	AUC
2-Hydroxyglutaric acid	Organic Acids	0.0012	1.1701	1.8734	0.964
Fumaric acid	Organic Acids	0.0451	0.9108	1.4587	0.804
Ketoleucine	Organic Acids	0.0213	0.744	1.8779	0.830
Maleic acid	Organic Acids	0.0369	0.8149	1.563	0.848
Oxoglutaric acid	Organic Acids	0.0205	1.8013	1.4442	0.839
Butyric acid	SCFAs	0.0041	1.1221	1.9293	0.875
Isobutyric acid	SCFAs	0.0140	1.8177	1.9302	0.920
Isovaleric acid	SCFAs	0.0093	3.0199	1.9011	0.893
Propanoic acid	SCFAs	0.0140	1.2655	2.0848	0.929
L-Glutamic acid	Amino Acids	0.0003	1.2001	2.166	0.938
L-Glutamine	Amino Acids	0.0074	−0.4505	1.5583	0.866
Pyroglutamic acid	Amino Acids	0.0003	1.4647	2.1692	0.920
Ornithine	Amino Acids	0.0022	1.011	1.9619	0.929
2-Methylhexanoic acid	Fatty Acids	0.0401	0.8945	1.5612	0.830
Ethylmethylacetic acid	Fatty Acids	0.0140	3.453	1.886	0.866
Methylsuccinic acid	Fatty Acids	0.0093	1.1711	2.0151	0.857
4-Hydroxyphenylpyruvic acid	Benzenoids	0.0080	1.862	1.7764	0.955
Benzenebutanoic acid	Benzenoids	0.0093	1.3484	2.1129	0.875
Phenylpyruvic acid	Benzenoids	0.0157	0.3421	1.9231	0.839

**FIGURE 4:**
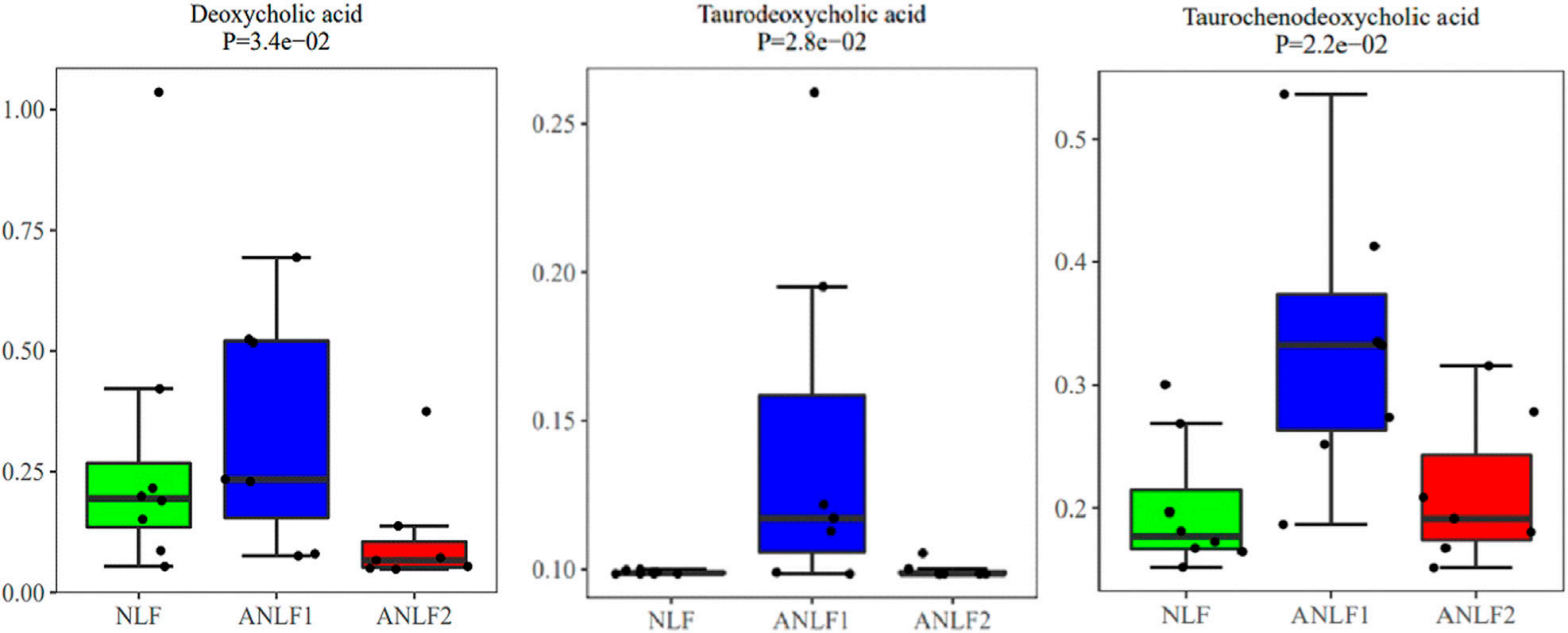
Box plots of the three different metabolites between the ANLF1 and ANLF2 group.

In addition, the differential metabolic components of ANLF1 and ANLF2 with different degrees of abnormal liver function were further analyzed. Only three bile acid metabolites, namely, taurodeoxycholic acid, taurochenodeoxycholic acid, and deoxycholic acid, were significantly different. As indicated in Figure 4, the contents of these three bile acid metabolites increased at first and then decreased with the aggravation of liver injury. Therefore, it was presumed that the early alterations of bile acids in VPA therapy should be paid much more attention.

### 3.3 Mechanistic evaluation of SIRT1/FXR signaling pathway

According to the findings of metabolomics above, we further explored the possible mechanism of VPA-induced liver dysfunction based on the bile acid metabolic pathway.

#### 3.3.1 VPA-induced HepG2 cell damage

**FIGURE 5:**
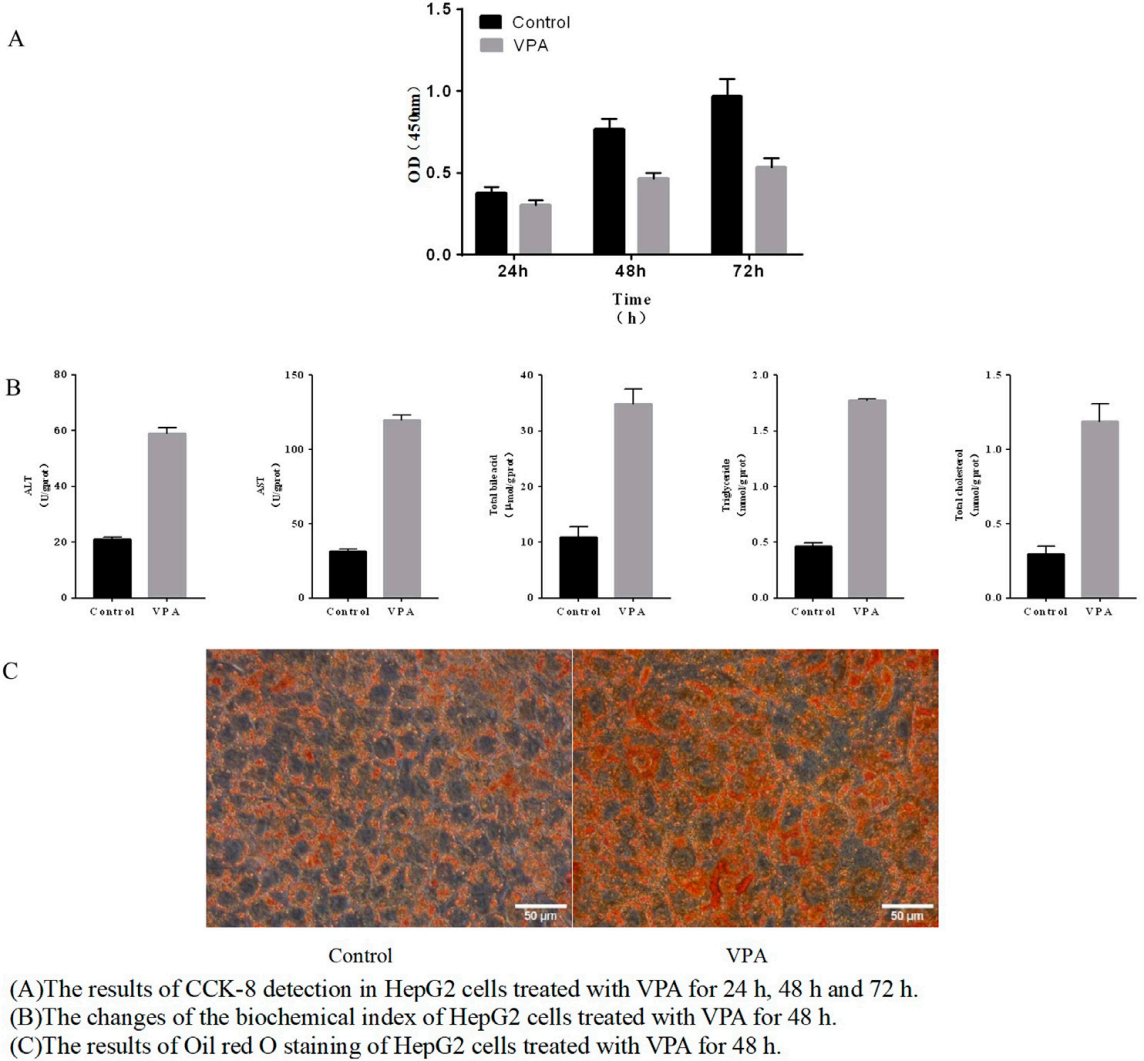
VPA-induced HepG2 cell damage **(A)** The results of CCK-8 detection in HepG2 cells treated with VPA for 24 h, 48 h and 72 h. **(B)** The changes of the biochemical index of HepG2 cells treated with VPA for 48 h. **(C)** The results of Oil red O staining of HepG2 cells treated with VPA for 48 h.

To examine cellular toxicity effects induced by VPA, an CCK8 assay was performed with VPA (2 mM) applied to HepG2 cells for 24 h, 48h and 72 h respectively. As shown in Figure 5A, it was indicated that VPA can inhibit the proliferation of HepG2 cells, and the degree of inhibition deepens with the extension of treatment time.

After treating HepG2 cells for 48 h, ALT and AST enzyme activities in the cells were significantly increased, and total bile acid, triglyceride and cholesterol content were significantly increased (*p* < 0.05), and the results are shown in Figure 5B. Further, the results shown in Figure 5C of oil red O staining indicated that the red intracellular lipid droplets increased significantly after VPA treatment, suggesting an increase in intracellular lipid content, which was consistent with the aforementioned results of triglyceride content determination. The combined biochemical index examination results and the results of oil red O staining indicated that VPA had a certain toxic effect on HepG2 cells.

#### 3.3.2 Expression of genes and proteins related to bile acids

**FIGURE 6:**
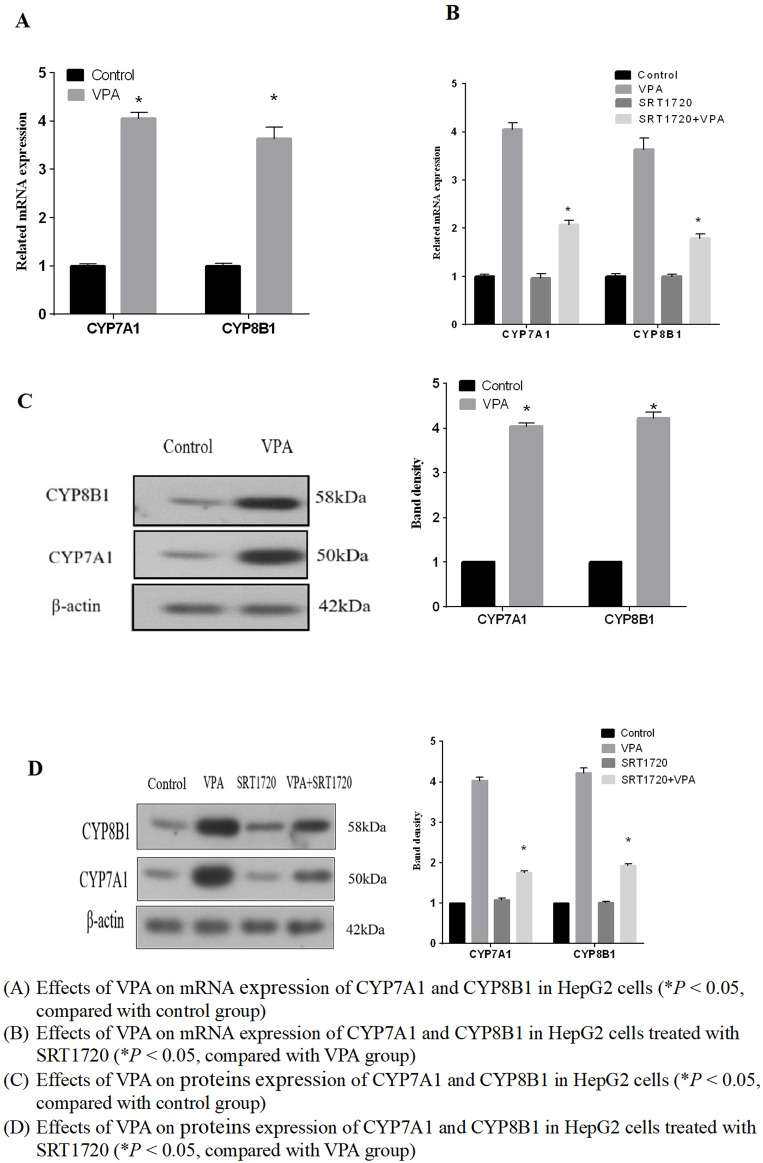
Effects of VPA on mRNA and expression of proteins of CYP7A1 and CYP8B1 **(A)** Effects of VPA on mRNA expression of CYP7A1 and CYP8B1 in HepG2 cells (*P < 0.05, compared with control group). **(B)** Effects of VPA on mRNA expression of CYP7A1 and CYP8B1 in HepG2 cells treated with SRT1720 (*P < 0.05, compared with VPA group). **(C)** Effects of VPA on proteins expression of CYP7A1 and CYP8B1 in HepG2 cells (*P < 0.05, compared with control group). **(D)** Effects of VPA on proteins expression of CYP7A1 and CYP8B1 in HepG2 cells treated with SRT1720 (*P < 0.05, compared with VPA group).

In the previous experimental studies of our research group, it was found that the important synthetases CYP7A1 and CYP8B1 in the classical synthetic pathway of bile acid metabolic pathway changed after VPA treatment. However, the changes of CYP7A1 and CYP8B1 were inconsistent in animal experiments and cell experiments. Therefore, we mainly investigated the expression of CYP7A1 and CYP8B1 in this part. The results showed that the mRNA and protein expressions of CYP7A1 and CYP8B1 were upregulated (*p* < 0.05), as shown in Figures 6A, C, indicating that VPA can increase the synthesis of bile acids in the classical pathway. It also explained the phenomenon of bile acid elevation in the patients with mild liver dysfunction to some extent.

#### 3.3.3 The SIRT1/FXR pathway participates in VPA-induced liver injury

**FIGURE 7:**
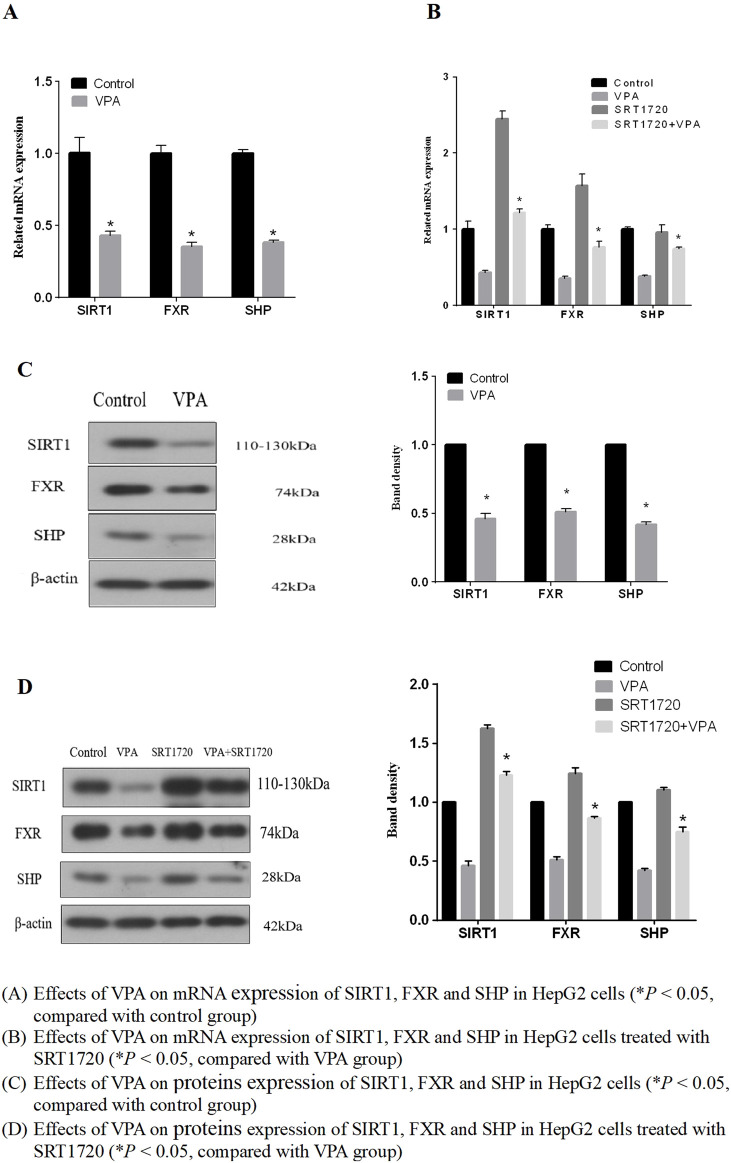
Effects of VPA on mRNA and expression of proteins of SIRT1, FXR and SHP. **(A)** Effects of VPA on mRNA expression of SIRT1, FXR and SHP in HepG2 cells (*P < 0.05, compared with control group). **(B)** Effects of VPA on mRNA expression of SIRT1, FXR and SHP in HepG2 cells treated with SRT1720 (*P < 0.05, compared with VPA group). **(C)** Effects of VPA on proteins expression of SIRT1, FXR and SHP in HepG2 cells (*P < 0.05, compared with control group). **(D)** Effects of VPA on proteins expression of SIRT1, FXR and SHP in HepG2 cells treated with SRT1720 (*P < 0.05, compared with VPA group).

We further investigated the effect of VPA on SIRT1 expression as the results shown in Figures 7A, C, indicating that the mRNA and protein expressions of SIRT1, FXR and their downstream target protein SHP were downregulated after VPA treatment (*p* < 0.05). Hence, it can be supposed that SIRT1 may be the upstream signaling pathway of FXR and participate in VPA-induced liver injury.

Based on the results that VPA can inhibit the expression of SIRT1 and FXR, we further explored whether the activation of SIRT1 has a protective effect on VPA-induced liver injury. As the results shown in Figures 7B, D, it can be seen that the synthetic SIRT1 agonist SRT1720 could alleviate the inhibition of VPA on the proliferation of HepG2 cells. Real-time PCR and Western blot results showed that the mRNA and protein expressions of SIRT1, FXR and SHP could be increased after treatment with 5 μM SRT1720 (*p* < 0.05), and the downregulation of SIRT1 and FXR induced by VPA could be reduced after pretreatment with SRT1720. In addition, SRT1720 activation of SIRT1 can reduce the upregulation of CYP7A1 and CYP8B1 mRNA and protein expression by VPA (Figures 6B, D), suggesting that SIRT1 may be a potential intervention target to alleviate VPA-induced liver injury.

**FIGURE 8:**
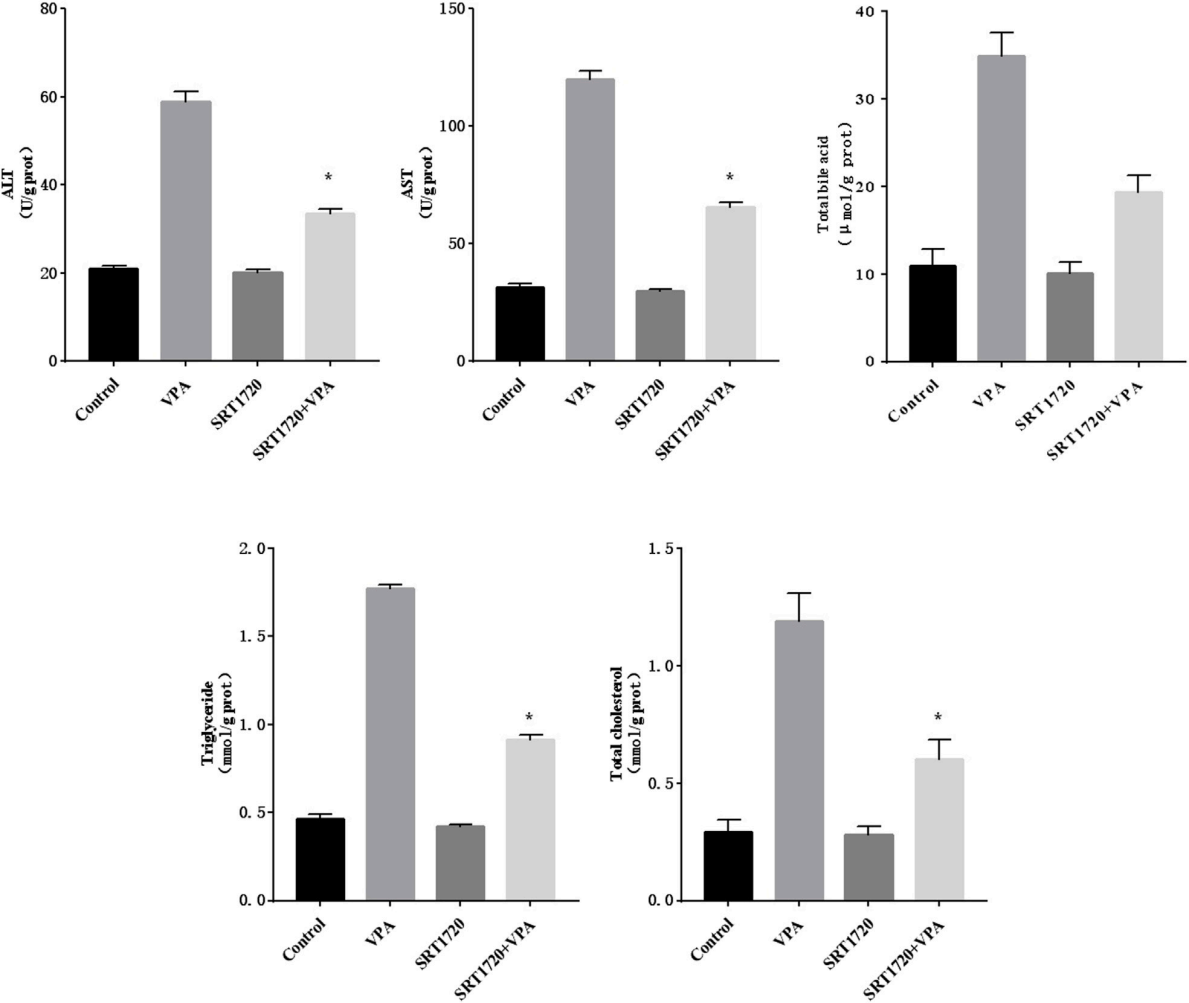
The changes of the biochemical index of HepG2 cells treated with SRT1720.

Further, various biochemical indicators (ALT, AST, total bile acid, triglyceride and cholesterol) were measured to observe the abnormal changes in liver function after SRT1720 activation. The results were shown in Figure 8, indicating that SRT1720 activation could alleviate the severity of liver function abnormalities induced by VPA.

**FIGURE 9:**
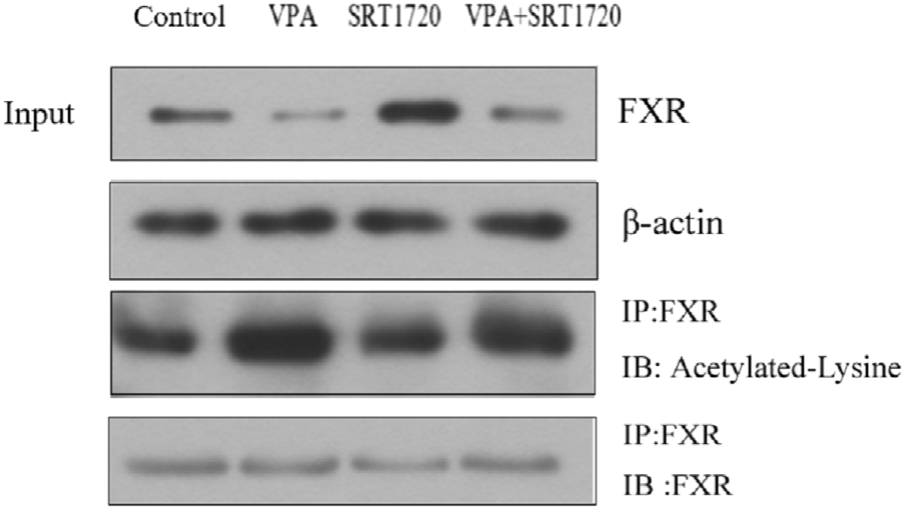
Effect of VPA on the acetylation level of FXR in HepG2 cells.

Since the activity of FXR is related to its acetylation level, and SIRT1 participated in maintaining the dynamic balance of FXR acetylation, the effect of VPA on the acetylation level of FXR was further investigated by immunocoprecipitation. The results in Figure 9 showed that VPA significantly increased the acetylation level of FXR, and the application of agonist SRT1720 to pretreat HepG2 cells can antagonize the acetylation of FXR by VPA to a certain extent. Therefore, we speculated that SIRT1 may be the upstream signaling pathway of FXR.

## 4 Discussion

Drug-induced hepatotoxicity poses a remarkable challenge for drug development and pharmacotherapy. As a widely used antiepileptic drug, VPA requires a long-term prescription to treat the nervous system diseases. Therefore, VPA-induced hepatotoxicity has caused extensive concern in the medical and pharmacological fields (Nanau and Neuman, 2013; Star et al., 2014). Although a large number of cases of VPA-induced hepatotoxicity have been reported (Gayam et al., 2018; Bassett et al., 2019; Gerstner et al., 2007), its exact molecular mechanism has not yet been identified. At present, most of the studies on VPA-induced liver injury focus on animals and cells or on a certain aspect of the mechanism, and these results can only partially explain the cause of liver injury. Moreover, it is difficult to predict occurrence of liver injury in advance (Lheureux and Hantson, 2009; Li et al., 2017; Bai et al., 2017). It has been reported that the early diagnosis and prompt withdrawal of VPA may lead to clinical improvements in VPA-induced hepatotoxicity (Gayam et al., 2018). Therefore, exploring early warning markers of liver injury is of great clinical significance. It has been demonstrated that MS-based metabolomics is a powerful tool to profile the changes of endogenous metabolites, providing a new method for finding markers and a deeper insight into the underlying mechanistic abnormalities (Nicholson et al., 2002; Zhang et al., 2012). In recent years, researchers have devoted great attention to exploring the markers of VPA-induced liver injury by metabolomics (Lee et al., 2009; Huo et al., 2014). However, few studies have focused on epileptic children, who are at a high risk of liver injury, especially those younger than 2 years of age, with a risk of one in 500. To the best of our knowledge, this is the first study involving pediatric patients with liver failure undergoing VPA treatment at two different degrees of liver function.

At present, although non-targeted metabolomics has been widely used due to its high throughput characteristics, it still has certain drawbacks. The specificity of non-targeted methods is relatively poor, and the results are easily affected by instruments and other external factors. Moreover, most of the results of non-targeted metabolomics are in the enrichment of the pathways, making it difficult to obtain accurate differential metabolic components. On the other hand, targeted metabolomics technology can be used for purposeful examination according to the molecular of known substances, and the accuracy and specificity are greatly improved, especially when the sample size is relatively small, as in this study. Therefore, this broad-spectrum targeted quantitative method, which can simultaneously detect 310 small molecular metabolites with high throughput and absolute quantification, was employed to investigate the metabolomic profiles among the three groups in terms of the dynamic change of VPA-induced liver injury.

A total of 19 significantly changed metabolites were identified after the data processing and statistical analysis. Four fatty acids (butyric acid, isobutyric acid, isovaleric acid, and propanoic acid) were elevated significantly with the severity of liver injury, indicating the inhibitory effect of VPA on fatty acid metabolism. These results were consistent with previous reports that VPA inhibits fatty acid metabolism by exhausting acetyl-CoA and carnitine and inhibiting the mitochondrial β-oxidation pathway (Guo et al., 2019; Lheureux and Hantson, 2009).

A further investigation of the difference of metabolic components between the two groups with different degrees of abnormal liver function revealed that only three kinds of bile acids were significantly different. At first, the contents of the three bile acids increased significantly in the mild liver injury group, which may be due to the inhibition of VPA on the negative feedback regulation of bile acid metabolism signal path-way, leading to the increase of bile acids synthesis with cholestasis in the initial stage. As the bile acids increased, the liver injury grew much more serious, decreasing the liver’s ability to synthesize the bile acids. Accordingly, the content of the three bile acids decreased in serious liver injury, consistent with the research of Chen et al. in animals (Chen et al., 2019). This dynamic process of bile acids may be the unique characteristic of VPA-induced liver injury, indicating that early alterations of bile acids in VPA therapy should be paid much more attention. Thus, the mechanism of VPA-induced liver injury based on the bile acid metabolic pathway needs to be further studied.

According to the results obtained from the metabolomics research, we further focused on the possible mechanism of VPA-induced hepatotoxicity involved in the bile acid metabolic pathway. As a major receptor of bile acid, FXR plays a crucial role in hepatic bile acid synthesis, metabolism, and transport (Russell, 2003; Ferrebee and Dawson, 2015; Cariello et al., 2018). FXR has been reported as a possible target protein that mediates drug-induced liver injury, thus, its activation may be helpful in reducing or reserving injury (Jin et al., 2015; Gai et al., 2020). However, there are no researches reported how VPA affects FXR-mediated bile acid-related pathways. Chen et al. in our team has reported that VPA can downregulate the expression of FXR, but activation of FXR by its agonist GW4064 could not lighten the inhibition on target genes of VPA (Chen et al., 2019).Therefore, we presumed that there may be some other upstream regulatory factors involved.

It has been found that FXR activity is related to its acetylation level, which, in turn, is dynamically regulated by p300 and silent information regulator1(SIRT1), a class III NAD^+^-dependent histone deacetylase (HDAC) (Kemper et al., 2009; Fang et al., 2008). Moreover, VPA was proven to inhibit HDAC and has become an adjuvant drug for tumor therapy in recent years (Berendsen et al., 2019; Aztopal et al., 2018). Consequently, we hypothesized that VPA suppressed the activity of SIRT1 by inhibiting the activity of HDAC, interfering with the dynamic balance of the acetylation modification of FXR, and then inhibiting the effect of FXR, leading to the perturbation of bile acid homeostasis and eventually liver injury. Also, the results confirmed the hypothesis that VPA affected the deacetylation modification of FXR by inhibiting the activity of SIRT1, which leaded to the increase of the acetylation level of FXR, and further inhibited FXR’s regulation of downstream target genes. These results indicated that SIRT1-FXR signaling pathway plays an important role in VPA-induced liver dysfunction, and it is possible to search for protective agents for liver dysfunction in this signaling pathway for follow-up studies.

This is the first study to report that the SIRT1/FXR pathway may participate in VPA-induced hepatotoxicity due to the observed changes of the bile acid metabolic pathway found in the metabolomics research. These results of the study could offer greater comprehension of a potential mechanism associated with VPA-induced hepatotoxicity and provide potential targets that could be applied to prevent VPA-induced hepatotoxicity. Nevertheless, further studies are needed to evaluate the role of SIRT1 in VPA-induced liver dysfunction from the perspective of the SIRT1/FXR signal pathway. In addition to directly upregulating the acetylation level of FXR, it was reported that SIRT1 may also indirectly regulate FXR activity through hepatocyte nuclear factor 1α (HNF1α). Purushotham et al. reported that deficiency of SIRT1 in the liver decreased HNF1α recruitment to the FXR promoter and reduces the expression of FXR, resulting in abnormal bile acid metabolism (Purushotham et al., 2012). In addition, it was shown that both acetylation and SUMOylation of FXR occur on lysine residues and interact with each other. Aspergillus A, which is also an HDACs inhibitor like VPA, was proved to upregulate PXR acetylation and SUMOylation at the same time (Balasubramaniyan et al., 2013). Therefore, whether VPA will affect SUMOylation while upregulating FXR acetylation through SIRT1 is also a question worthy of further exploration.

This study also has some limitations that should be considered. To eliminate the influence of drug combinations, all the cases enrolled in this study were children with epilepsy who were treated with VPA as a monotherapy, therefore, VPA-induced hepatotoxicity incidence was very low in these subjects. Moreover, the number of cases included in this study was relatively small, and a follow-up study was needed to expand the sample size to confirm the results. Due to the limitation of sample size, it is difficult to clarify the definite biomarkers. Therefore, multicenter research with large sample size needs to be carried out in the future.

## 5 Conclusion

This study systematically investigated how endogenous metabolic components change with the development of liver injury in children exhibiting VPA-induced hepatotoxicity using a full quantitative targeted metabolomics approach. A total of 195 metabolic components were quantitatively analyzed and 19 identified metabolites showed a strong association with VPA-induced hepatotoxicity, including five organic acids, four short-chain fatty acids, four amino acids, three fatty acids, and three benzenoids. Moreover, only three bile acid metabolites, namely, taurodeoxycholic acid, taurochenodeoxycholic acid, and deoxycholic acid, were significantly different between the ANLF1 and ANLF2 groups with different degrees of abnormal liver function. In addition, based on the results of the metabolomics research, it was the first time to clarify that the SIRT1/FXR pathway participated in VPA-induced hepatotoxicity, providing new insights into the hepatotoxicity mechanisms of VPA. Taken together, this metabolomic research could offer a greater comprehension of the dynamic change of VPA-induced hepatotoxicity and promote a further understanding of its mechanisms.

## Data Availability

The raw data supporting the conclusions of this article will be made available by the authors, without undue reservation.
