# Supplementary material for: The role of SIRT1-FXR signaling pathway in valproic acid induced liver injury: a quantitative targeted metabolomic evaluation in epileptic children
Source: Front Pharmacol. 2024 Nov 7;15:1477619. doi: 10.3389/fphar.2024.1477619 (PMC11578826; doi:10.3389/fphar.2024.1477619)
Supplement: Supplementary file 1 [file Table1.DOCX]

Table S1. The primer sequences used for real-time PCR

| Gene name | Sequence (5’-3’) | Product length (bp) |
| --- | --- | --- |
| CYP7A1 | Forward: GCTTGAGGCACGAGAACC  Reverse: CAGAAAGTCGCTGGAATG | 166 |
| CYP8B1 | Forward: GTTTGTCTACTCCCTGCTGT  Reverse: CTGATGCCCTCCTTCTCC | 111 |
| FXR | Forward: GATTTCCTCGTCATCCTA  Reverse: GAAGAAACCTTTACACCC | 274 |
| SIRT1 | Forward: TATCCTTTCAGAACCACCAA  Reverse: CGAGCATAAATACCATCCCT | 168 |
| SHP | Forward: GGAATATGCCTGCCTGAAAG  Reverse: CCAATGATAGGGCGAAAGAA | 234 |
| β-actin | Forward: GGCACCCAGCACAATGAA  Reverse: TAGAAGCATTTGCGGTGG | 168 |


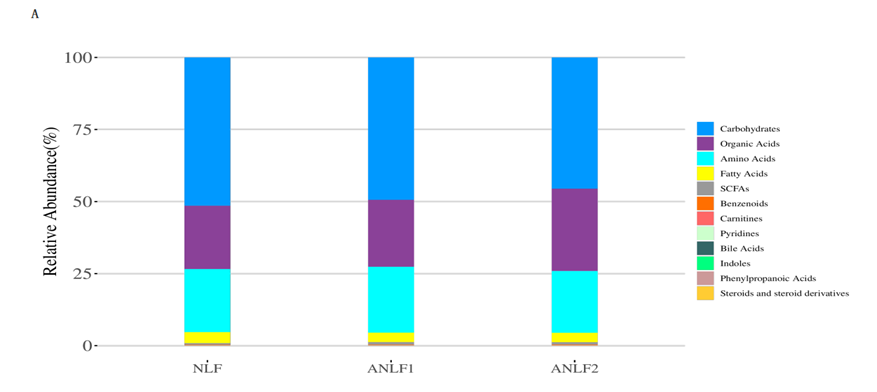


Figure S1 The relative abundance histogram of the endogenous metabolites in each group


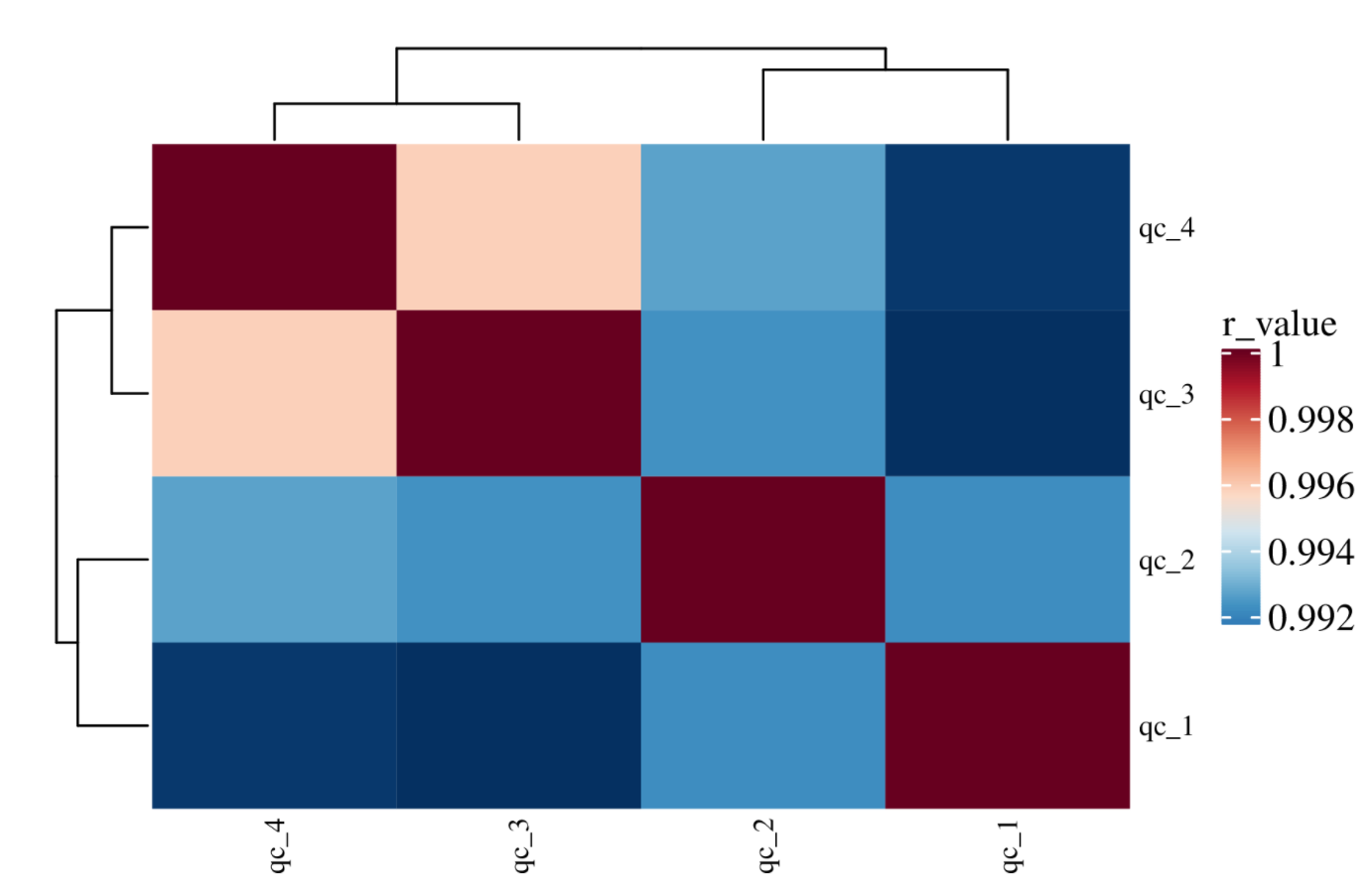

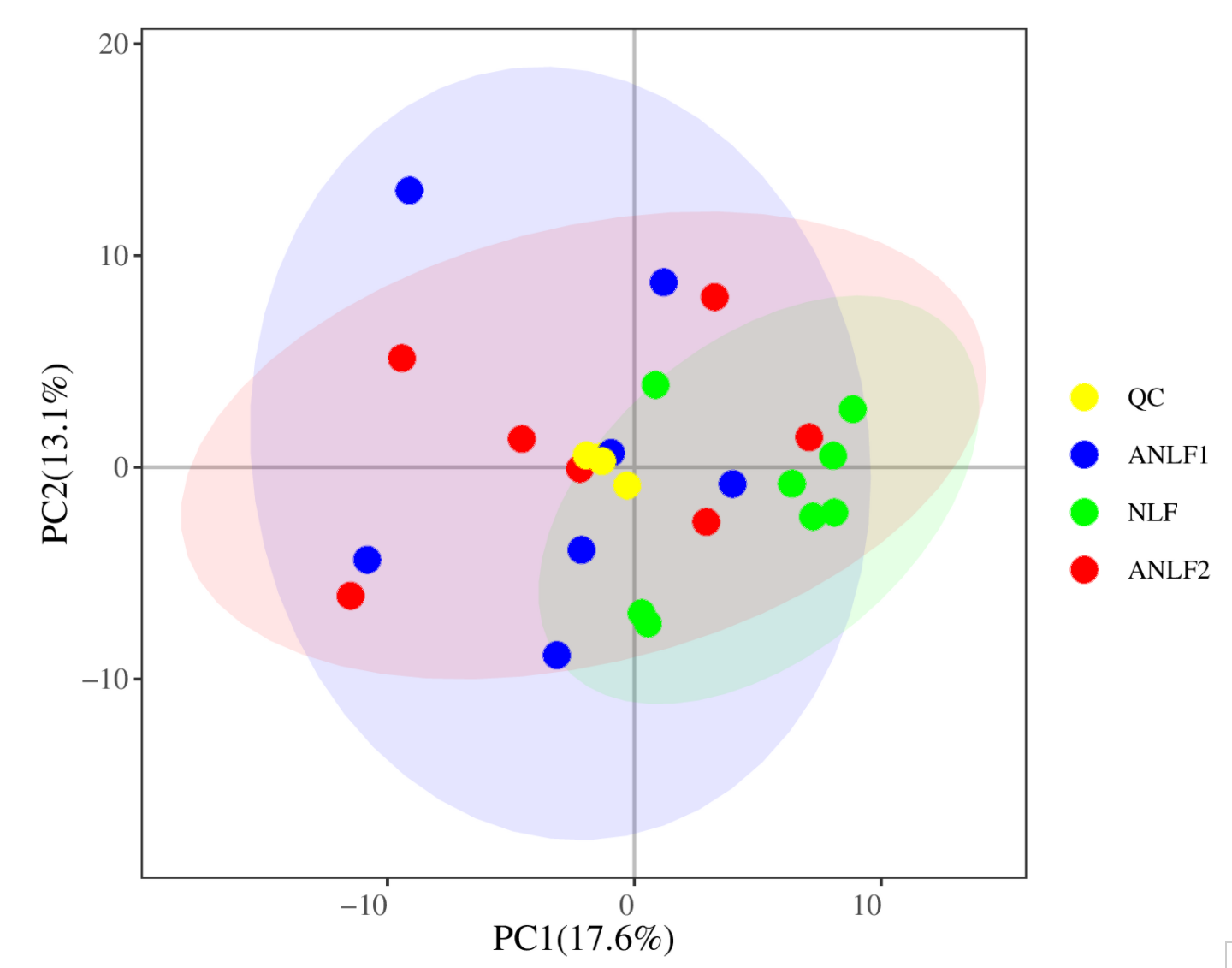


A

B

Figure S2 The heat map (A) and plot of PCA (B) of the QC samples


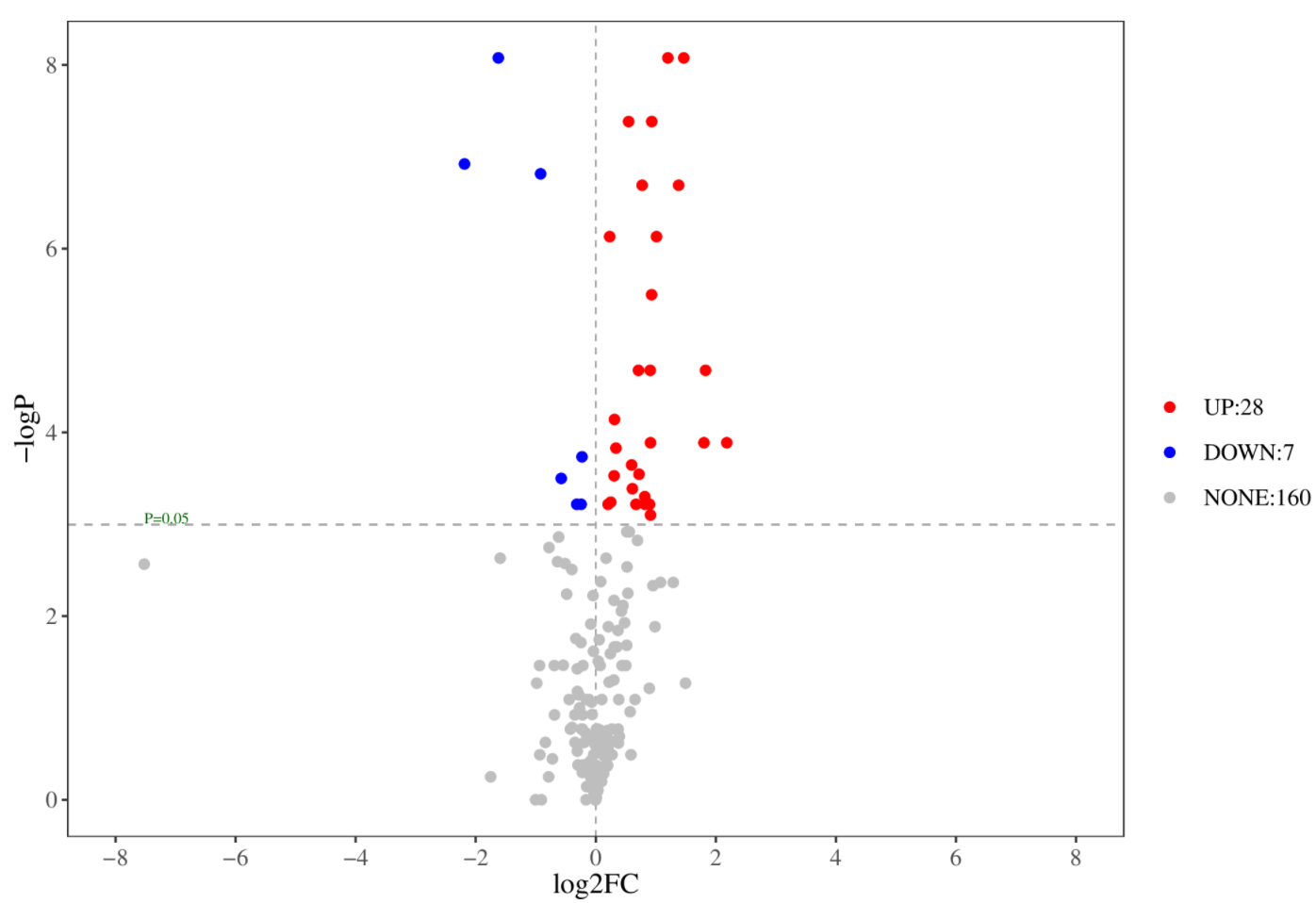

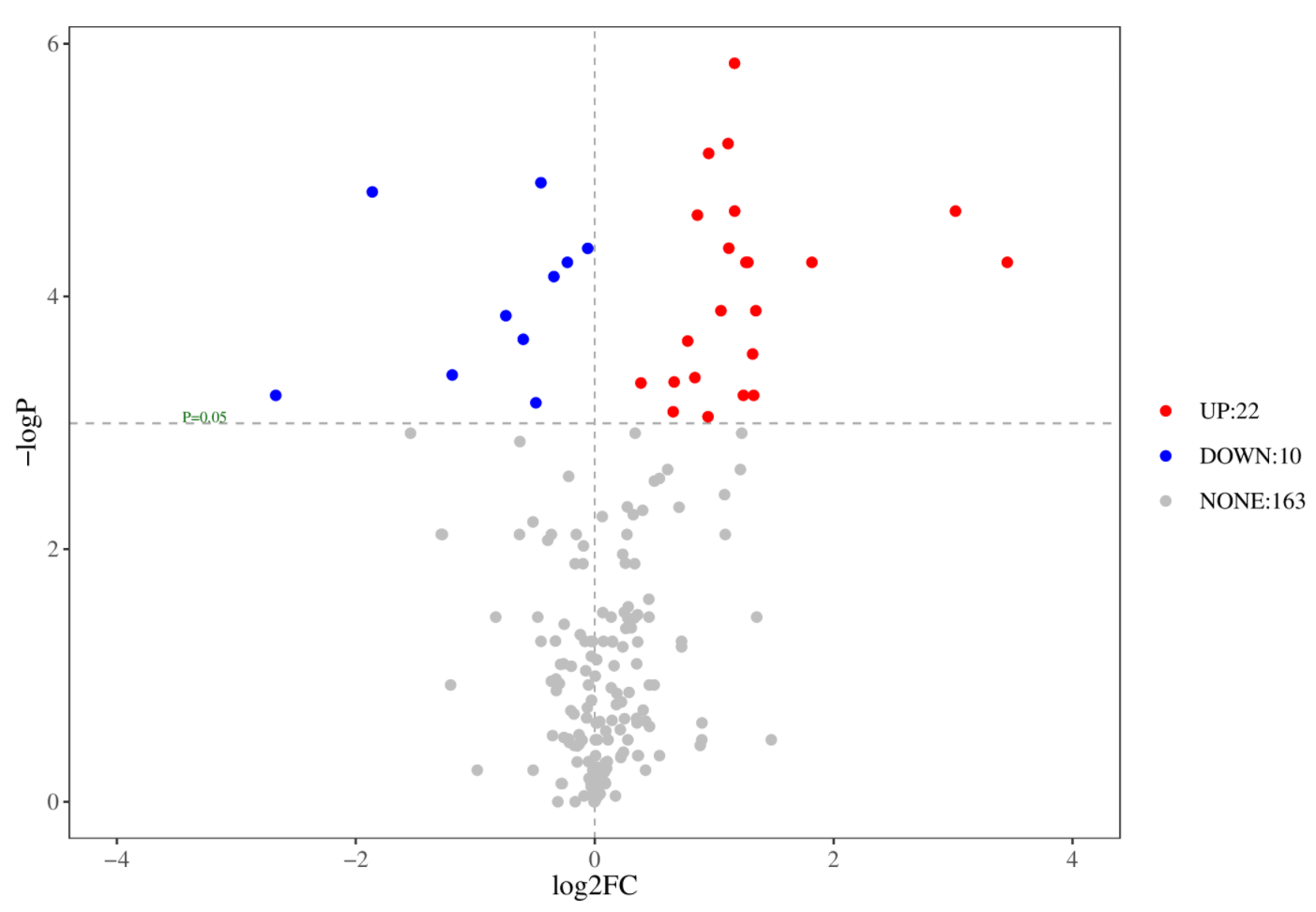

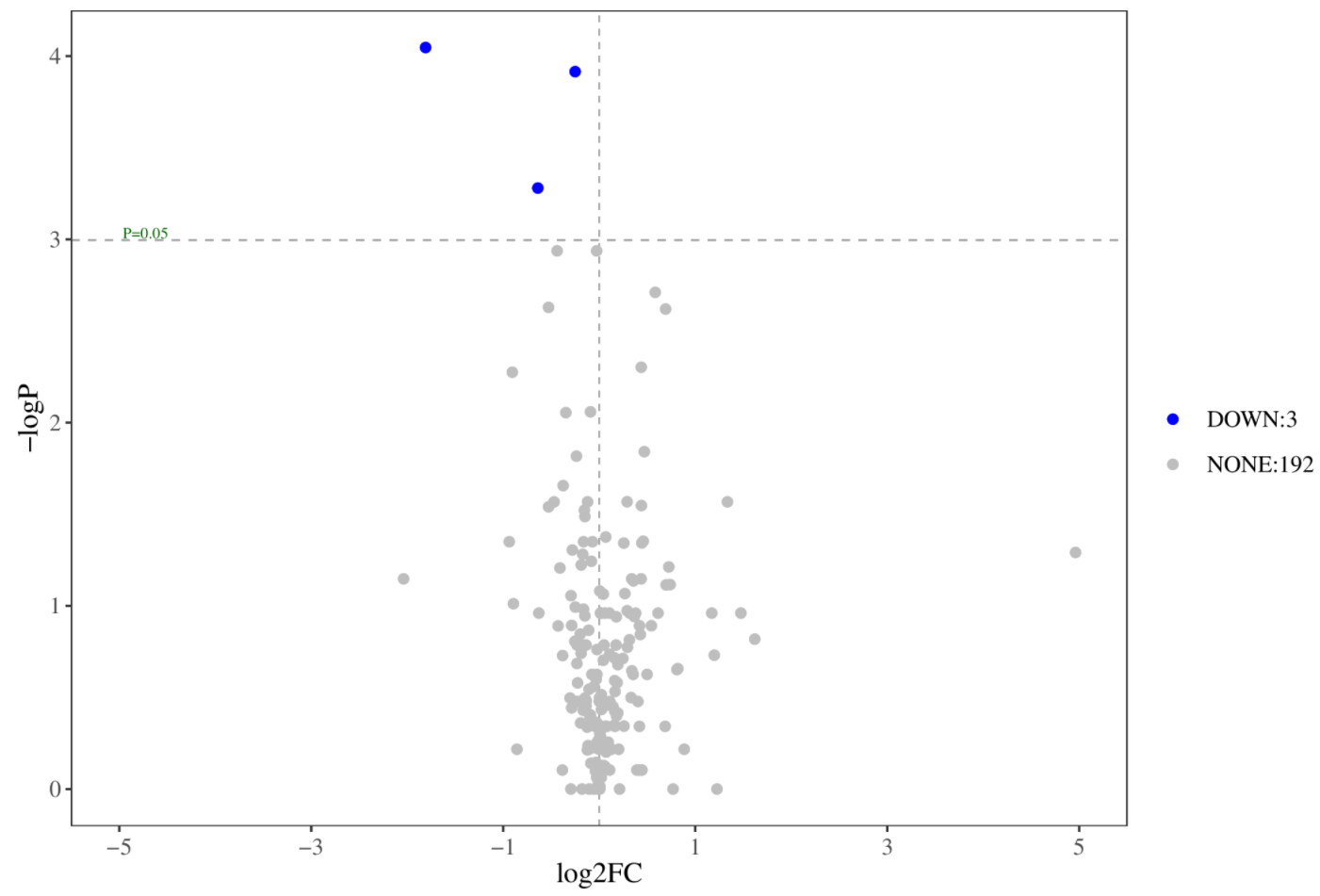


ANLF1 vs. ANLF2

NLF vs. ANLF1

NLF vs. ANLF2

Figure S3 Volcano plots of the serum of children with epilepsy between the ANLF1 or ANLF2 group and NLF group
